# Signal-to-signal neural networks for improved spike estimation from calcium imaging data

**DOI:** 10.1371/journal.pcbi.1007921

**Published:** 2021-03-01

**Authors:** Jilt Sebastian, Mriganka Sur, Hema A. Murthy, Mathew Magimai-Doss

**Affiliations:** 1 Idiap Research Institute, Martigny, Switzerland; 2 Department of Computer Science and Engineering, Indian Institute of Technology Madras, Chennai, India; 3 Department of Brain and Cognitive Sciences, Massachusetts Institute of Technology Cambridge, Massachusetts, United States of America; University of Geneva, SWITZERLAND

## Abstract

Spiking information of individual neurons is essential for functional and behavioral analysis in neuroscience research. Calcium imaging techniques are generally employed to obtain activities of neuronal populations. However, these techniques result in slowly-varying fluorescence signals with low temporal resolution. Estimating the temporal positions of the neuronal action potentials from these signals is a challenging problem. In the literature, several generative model-based and data-driven algorithms have been studied with varied levels of success. This article proposes a neural network-based signal-to-signal conversion approach, where it takes as input raw-fluorescence signal and learns to estimate the spike information in an end-to-end fashion. Theoretically, the proposed approach formulates the spike estimation as a single channel source separation problem with unknown mixing conditions. The source corresponding to the action potentials at a lower resolution is estimated at the output. Experimental studies on the spikefinder challenge dataset show that the proposed signal-to-signal conversion approach significantly outperforms state-of-the-art-methods in terms of Pearson’s correlation coefficient, Spearman’s rank correlation coefficient and yields comparable performance for the area under the receiver operating characteristics measure. We also show that the resulting system: (a) has low complexity with respect to existing supervised approaches and is reproducible; (b) is layer-wise interpretable, and (c) has the capability to generalize across different calcium indicators.

## Author summary

Information processing by a population of neurons is studied using two-photon calcium imaging techniques. A neuronal spike results in an increased intracellular calcium concentration. Fluorescent calcium indicators change their brightness upon a change in the calcium concentration, and this change is captured in the imaging technique. The task of estimating the actual spike positions from the brightness variations is formally referred to as spike estimation. Several signal processing and machine learning-based algorithms have been proposed in the past to solve this problem. However, the task is still far from being solved. Here we present a novel neural network-based data-driven algorithm for spike estimation. Our method takes the fluorescence recording as the input and synthesizes the spike information signal, which is well-correlated with the actual spike positions. Our method outperforms state-of-the-art methods on a standard evaluation framework. We further analyze different components of the model and discuss its benefits.

## 1 Introduction

Analyzing the brain’s responses to several types of stimuli enables an understanding of brain behavior and cognition. The responses of the neurons manifest as a spike train, which encodes the information present in the stimulus. State-of-the-art scanning methods track the activity of a population of neurons by using fluorescence emitting capability of calcium indicator proteins/dye [1–4]. However, the calcium fluorescence recording of each neuron is only an indirect indicator of the actual spiking process. The presence of fluorescence level fluctuations, slow dynamics of the calcium fluorescence signal, and unknown noise-levels make it hard to identify the exact underlying spike information [5–7]. Hence, approaches capable of obtaining the spike positions from the calcium fluorescence signals are of utmost interest to computational neuroscience community.

We provide a brief overview of the existing spike estimation algorithms. They can be broadly categorized into generative and data-driven approaches:

1. Generative methods model the fluorescence signal as the response of the calcium indicator to the spike occurrences. They rely on several model-specific assumptions. Deconvolution-based approaches consider convolutive assumptions about the spiking process [8–11], whereas biophysical model-based approaches estimate the most probable spike train which generated the fluorescence output [12]. Other model-based approaches include template matching [3, 13], auto-regressive formulation [14] and, approximate Bayesian inference based on deconvolution [5, 15]. These models are limited by the apriori assumptions about the model, which has stringent approximations regarding the shape of the calcium response and the noise statistics. Recently, a non-model based signal processing approach for spike estimation is presented in [16] that uses the agnostic nature of group delay to estimate the spike locations. It has a comparable performance with other popular algorithms in the literature.
2. Supervised models predict the spike information from the fluorescence signal, either using a set of features derived from the signal or using the raw signal itself. Data-driven methods are recently gaining traction owing to the availability of simultaneous electrophysiological and two-photon scanning-based neuronal recordings. For instance, a neural network-based supervised Spike Triggered Mixture (STM) model [17] is used for learning the *λ* parameter of a given Poisson model in [18] to obtain the spike estimates from the calcium signals. Recent methods use fluorescence signals with or without a contextual window (supplementary material—[19]) for estimating the spike information. Neural network-based variants such as “convi6”, “Deepspike”, “Purgatorio”, and “Embedding of CNNs” have had varying levels of success and outperformed data-driven baseline method [17] on a standard evaluation framework (supplementary material—[19]). A gated recurrent unit (GRU)-based approach recently attempted to estimate down-sampled action potentials directly from the 2-D calcium imaging output, combining regions-of-interest (ROI) estimation and spike estimation tasks [20]. An adversarial variational autoencoder (VAE) is employed in [21] for improved spike inference compared to the factorized posteriors used in standard VAEs.

Spikefinder challenge (http://spikefinder.codeneuro.org/) [19], a contest aimed to standardize the spike estimation evaluation and improve the state-of-the-art, resulted in a new set of algorithms that performed better than the benchmark STM Model [17]. Although these algorithms use different techniques, no additional gain was obtained when the results of various techniques were combined. The objective of the challenge was to standardize the spike estimation evaluation and to provide a comparison of the state-of-the-art techniques. Most of the top-performing algorithms used convolutional (CNN), recurrent (RNN) and, deep neural networks (DNN) and its variants [19]. All the top-performing data-driven algorithms have a recurrent layer in the network and hence are computationally complex. The best-performing supervised model used a CNN architecture with an intermediate Long Short-Term Memory (LSTM) layer to predict the spiking probability from a contextual window of the fluorescence signal (“convi6” in the supplementary material of [19]). Generative models such as MLspike [12] and auto-regressive model presented in [14] were comparable to the supervised approaches when the dataset-specific parameter tuning was carried out on the training datasets. MLspike uses a biophysical model and estimates the most probable spike information given the fluorescence signal. The second-best generative approach in spikefinder [14] is based on an autoregressive approximation to the calcium fluorescence signal. Spike information is then estimated by solving a non-negative sparse optimization problem.

The spike estimation problem can also be formulated as a sequence-to-sequence modeling problem in the supervised learning paradigm. We succinctly describe the motivation and related works on sequence modeling to set the stage for the proposed spike estimation method. Approaches that model sequences in an end-to-end fashion are being developed recently for classification and regression tasks in audio processing and natural language understanding with commendable success. One of the first efforts in that direction is made in the context of machine translation [22]. This approach is later extended to speech recognition [23, 24]. Sequence-to-sequence models have been used for text summarization task using RNNs in [25] and for creating more accurate language models [26] in natural language processing. End-to-end methods have also been employed to predict the target speech from the overlapped speech mixture for speech separation task [27, 28], and clean speech for enhancement tasks [29, 30] in audio processing research. In this case, as the models are trained on raw-waveforms, they do not require handcrafted features. Instead, they learn the relevant information in a task-dependent manner from the signal directly. They also provide the flexibility to choose task-specific objective functions for training, which implicitly considers the temporal context. It is also possible to analyze the convolutional filters in the neural network to understand the learning trends in the temporal and frequency domains. Given that the spike train is also a temporal signal, it should be possible to formulate the spike estimation problem in an autoencoder framework.

**Fig 1:**
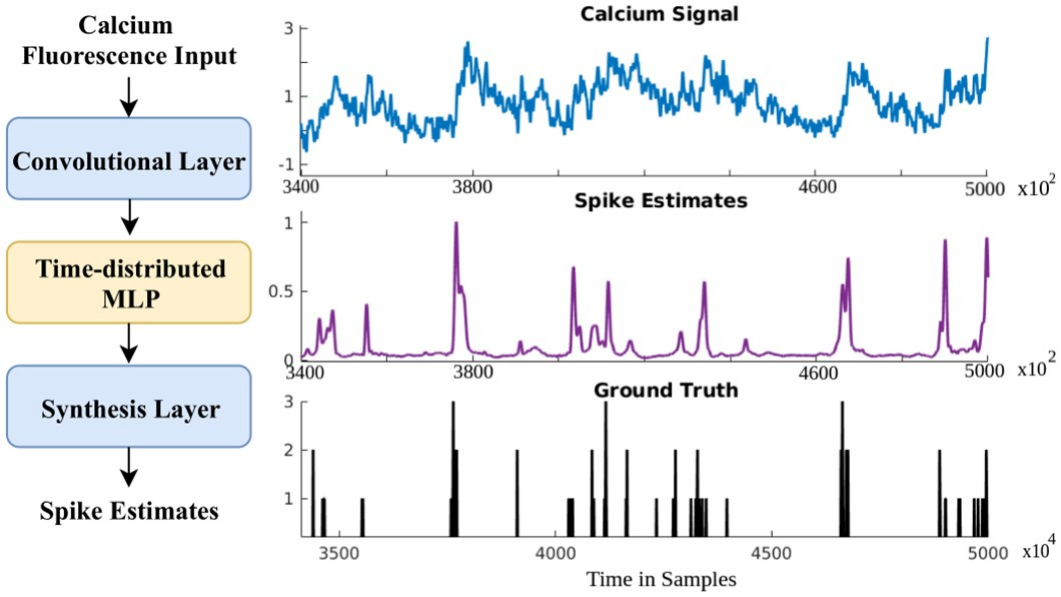
Signal-to-signal neural network (S2S). (***left***) Block diagram of the proposed approach. (***right***) An illustrative example calcium signal, its corresponding spike estimate, and the ground truth spike train.

Motivated by the success of the aforementioned sequence-to-sequence models, we present a signal-to-signal neural network (hereafter referred to as S2S) for the spike estimation task. The proposed method can be regarded as an analysis-synthesis method, where, as illustrated in Fig 1, the input calcium fluorescence signal is analyzed by an input convolution layer; filtered by time-distributed dense layer(s), also called as hidden layers (In this article, we use the terms dense layer and hidden layer interchangeably); and finally the spike signal is synthesized by an output convolution layer. The synthesis layer that generates signal samples makes our architecture distinct from other supervised spike estimation methods where only a single value is predicted/classified to at the output. All the network parameters are learned in an end-to-end manner in S2S with a cost function based on the Pearson’s correlation between the estimated spike signal and the ground truth spike signal. We hypothesize that such a spike estimation network can outperform existing approaches as it *reconstructs* the spike information for each input sample. This neural network differs from the sequence-to-sequence models in several aspects. First, the output signal’s nature or characteristics (discrete spike estimates) is very different from the input signal (calcium fluorescence trace). Second, it performs automatic short-time processing through shared weights across the temporal axis. Third, as we will see later (Section 2.4.4), each layer’s output can be visualized to gain insight into the information captured by each layer. The frequency responses of both analysis and synthesis layers in the network can also be analyzed.

The proposed S2S method is better than the competitive algorithms from the spikefinder contest with the same dataset and evaluation procedure. We also compare S2S with the best neural network-based model presented in the contest. We study the research questions in terms of reliability, generalization ability, dependency on training targets and, design concerns of S2S. In addition, we conduct a layer-wise analysis of the network to provide an intuitive explanation for the learning.

## 2 Results

### 2.1 Evaluation procedure

We used the Spikefinder challenge dataset [19] for evaluations. It consisted of five benchmarking datasets consisting of 92 recordings from 73 neurons. One part of this dataset was given for training the supervised models, and the other part for testing as a part of the competition. Five datasets [31] from GENIE project [32] were also available for training the models. Additional datasets were used to ensure that the supervised models do not over-fit the training data. For further details about the spike finder dataset, the reader is referred to [19]. As per the spike finder challenge protocol, we performed the training at 100 Hz and the evaluation at 25 Hz (40 ms bin width). The evaluation measures were Pearson’s correlation coefficient, Spearman’s rank correlation coefficient, and the area under the receiver operating characteristics (denoted as AUC).

We used Pearson’s correlation coefficient as the primary evaluation measure, as done in the spikefinder challenge. Rank (non-linear correlation) and AUC serves as secondary and tertiary evaluation measures, respectively. This standardization enabled us to benchmark the proposed S2S method against the challenge submissions. It is worth mentioning that the performance metrics were calculated solely based on the script provided by the spikefinder challenge organizers. The performance of the S2S method was compared to the top six algorithms in the spikefinder contest. They were based on either generative [12, 14] or supervised [17, 19, 33, 34] approaches, as discussed earlier (Lines 18-48). Seven out of the top-10 algorithms were deep learning-based supervised algorithms. Table 1 provides an overview of the baseline methods (taken from [19]).

**Table 1:** Overview of top-performing algorithms in spikefinder challenge.

Team	Contributor(s)	new	type	Model/Architecture
Team1	T. Deneux	No [12]	Generative	Biophysical model
Team2	N. Chenkov, T. McColgan	Yes	Supervised	conv / lstm
Team3	A. Speiser, J. Macke, S. Turaga	Yes	Supervised	RNN/CNN
Team4	P. Mineault	Yes	Supervised	residual / lstm
Team5	P. Rupprecht, S. Gerhard, R. W. Friedrich	Yes	Supervised	conv / max
Team6	J. Friedrich, L. Paninski	No [14]	Generative	Autoregressive model
Baseline: STM	L. Theis	No [17]	Supervised	DNN +Poisson model

### 2.2 Comparison to spikefinder algorithms

Table 2 compares the performance of the S2S method with the methods reported in the spikefinder challenge [19]. The S2S network outperformed all the state-of-the-art methods, both generative and supervised. It improved the test correlation by 46% compared to the best performing algorithm in the spikefinder contest. Change in the correlation with respect to the challenge baseline (denoted as Δ) was significantly high for the proposed approach (3.5 times compared to the best reference algorithm). S2S provided a relative improvement of 56% for the rank measure over the baseline with the best rank. It also had a similar AUC compared to the best method in terms of AUC measure. The deviation between the train and test sets’ correlation coefficient for the S2S method was small (0.0079). Interestingly, S2S is the only method for which the test set’s correlation coefficient was more than the training set, indicating that the proposed method generalizes well.

**Table 2:** Comparison of evaluation measures between S2S and state-of-the-art spikefinder baselines.

Team Name	Train correlation	Test correlation	Δ correlation	Rank	AUC
Team 1 MLspike new	0.4823	0.4382	0.0810	0.2878	0.846
Team 2 convi6	0.4727	0.4378	0.0806	0.3319	0.846
Team 3 DeepSpike	0.4730	0.4347	0.0775	0.3338	0.851
Team 4 Purgatorio	0.5370	0.4325	0.0753	0.3258	0.815
Team 5 Embedding of CNNs	0.4900	0.4291	0.0719	0.2822	0.821
Team 6 Suite2p	0.4752	0.4188	0.0617	0.3071	0.821
Baseline STM	0.4024	0.3572	-	0.2664	0.821
**Proposed** S2S	0.6325	**0.6404**	**0.2832**	**0.5208**	0.847

Spike trains were recorded originally at a very high sampling rate (10,000 Hz). The Spike train was down-sampled, and the calcium fluorescence signal was up-sampled to 100Hz for the experiments. Spike trains at 25 Hz (40 ms time-duration) can be effectively inferred via S2S, as seen from this evaluation. Since the resolution of the down-sampled spikes was much less than the original frequency, it can be interpreted as firing rate. S2S output closely follows the ground truth shape information and this resulted in a better correlation value.

### 2.3 Comparison to state-of-the-art supervised baseline

**Fig 2:**
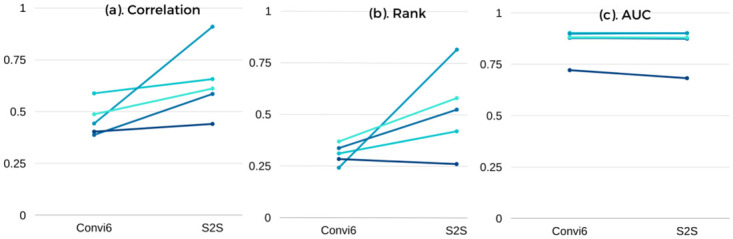
Dataset-wise performance to show the difference in evaluation measures between S2S and convi6. (a) Correlation measure, (b) Rank (non-linear correlation) measure and (c) AUC measure.

Fig 2 compares the performance of S2S with the best supervised baseline (Base) “convi6” (Second row in Table 2). The baseline approach (convi6) had the second position in the challenge, falling behind MLspike gracefully by 0.04%. The performance of S2S for each dataset of the spikefinder contest is shown in Table 3. S2S achieved significantly better Pearson’s and Spearman’s correlation values across all the five different test sets and achieved comparable AUC. We performed a paired t-test on the spikefinder test data to determine the statistical significance of dataset-wise results of S2S as compared to that of the convi6 model. Each test dataset was considered as a separate example file similar to that in the spikefinder challenge for the significance test. The null hypothesis (H) was that there is no difference in the performance metrics (correlation, rank, and AUC) between S2S and convi6, at 95% confidence level (*α* = 0.05). Table 4 shows the results of the statistical significance test for all of the evaluation measures. The t-value from the table for a degree of freedom four is found to be 2.132. The t-value 1 corresponds to the paired t-test between convi6 and S2S with Gaussian target (refer to Section 2.4.1 for details on the training target), and t-value 2 corresponds to the t-test between convi6 and S2S with the actual training target.

**Table 3:** Dataset-wise performance of S2S on the spikefinder test set.

Measure	Dataset 1	Dataset 2	Dataset 3	Dataset 4	Dataset 5	Average
Correlation	0.657	0.910	0.585	0.440	0.611	0.640
Rank	0.420	0.816	0.525	0.261	0.581	0.521
AUC	0.901	0.901	0.874	0.682	0.879	0.847

**Table 4:** Results on paired t-test for statistical significance. “H” represents the hypothesis that there is no difference in performance between S2S and convi6.

Measure	t-value 1	t-value 2	Accept/Reject H
Correlation	2.32	2.33	Reject
Rank	2.14	2.16	Reject
AUC	1.11	1.00	Accept

There was a significant improvement in the correlation and rank measures. This was further confirmed by the values of the mean and the standard deviation of the baseline methods, where the primary evaluation measure was correlation. The value of primary evaluation measure (0.640) was greater than the mean and two times the standard deviation (Mean +2× STD) of the baseline approaches (0.479), by 16.1%.

Scalability of the proposed approach was evaluated by choosing a higher resolution (100 Hz). It was observed that correlation measures are affected, while there was a graceful degradation for AUC measure. Similar performance degradations were observed for baseline methods as well (Table 5). We observed that the correlation measures were still better than all other algorithms evaluated at 25 Hz.

**Table 5:** Results on scalability experiments at 25 Hz and 100 Hz sampling rates.

Approach	Evaluation Sampling Rate	Correlation	Rank	AUC
S2S	25 Hz	0.640	0.522	0.848
S2S	100 Hz	0.527	0.448	0.846
Convi6	25 Hz	0.461	0.309	0.845
Convi6	100 Hz	0.367	0.181	0.844

We observed that the relative amplitude values of the spike estimate were closer to the ground truth as compared to that of the baseline algorithms (refer to Section 3.1). This was reflected in a large dynamic range for spike estimation values, which were also proportional to the ground truth. This suggests that it might be possible to threshold the spike estimates to get the spike train directly.

We investigated the retraining of the neural networks (new initialization and training on the same dataset) in the S2S method and compared with the convi6 method. We found that the S2S method was insensitive to re-initialization, while convi6 method performance varied on average (standard deviation of 5%, 8%, and 1% absolute for correlation, rank, and AUC, respectively). These results show that the proposed S2S method yields reliable estimates of the spike signal. The best baseline performance (for primary evaluation measure) was observed when using the weights provided by the spikefinder [19]. S2S was significantly faster than the baseline. On Sun GPU clusters, S2S training was 50 times faster than that of the baseline model. Other methods such as MLspike (winner of the spikefinder competition) and Vogelstein are based on signal processing and do not require training. The inference time of the neural network-based methods (convi6, S2S, and others) was *O*(*n*). Both the Vogelstein and STM (test) ran in linear time. MLspike took *O*(*nlogn*) for dynamic programming and additional time to auto-calibrate its parameters.

### 2.4 Analysis

In this part of the article, we provide an analysis of the proposed S2S method in terms of architecture, training target, generalization capabilities and layer-wise interpretation of the trained network.

#### 2.4.1 Training target

**Fig 3:**
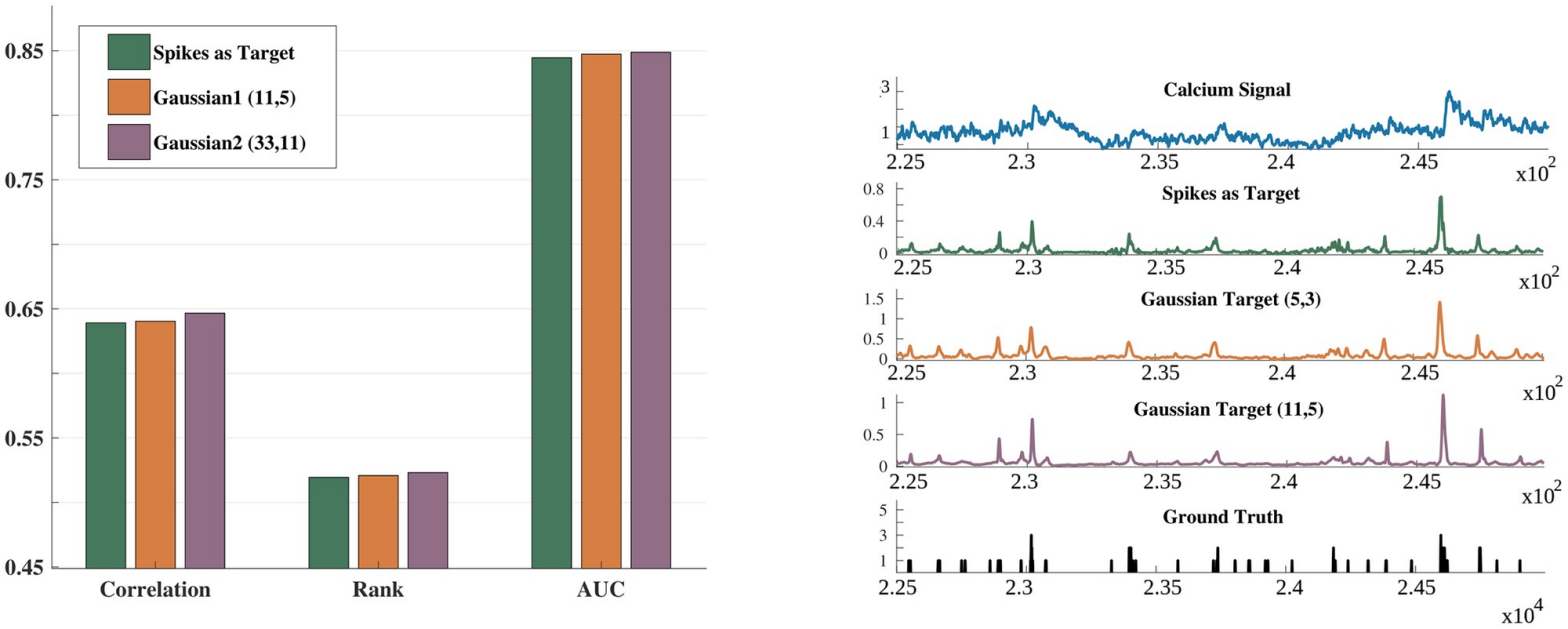
Change in the evaluation measures with Gaussian windowing of the training targets. (***left***) Bar diagram depicting the difference in Correlation, Rank and AUC when using the 100 Hz spike train as the target and when convolving this target with a Gaussian window to generate a smoothed training target. Two different windowing sizes are shown; 11 samples with 5 standard deviation and 33 samples with 11 standard deviation. (***right***) From top to bottom: An example calcium fluorescence signal and its corresponding training targets (spikes at 100 Hz, Gaussian targets with 5 and 11 samples each, and the ground truth at 10,000 Hz). Gaussian training targets are having an equivalent shape to the spikes target at 100 Hz. Note that all of them are approximations of the original ground truth.

As discussed in the Methods section (Section 4.1.2), the S2S network was trained with a modified ground truth or target by convolving the original discrete spike information with a Gaussian function. Although the Gaussian window hyper-parameters (11, 5), where (*x*, *y*) denotes Gaussian window width *x* and standard deviation *y* in the number of samples, were obtained through cross-validation during the network training, a question that arises is: what is the impact of less-sparse (more-dense) targets? So, we investigated the S2S network’s training by convolving the target spike signal with the Gaussian windows of variable sizes. For the sake of clarity, Fig 3 compares the performance obtained for (11, 5) with the performance obtained for (33, 11) and the performance with a discrete spike signal as the target signal. It can be observed that convolving with Gaussian function helps. The performance in terms of all three evaluation measures improves from a discrete spike signal to a Gaussian target. Increasing the width of the Gaussian window beyond (33, 11) resulted in reduced performance, as the shape of the training target became very different from the discrete target (actual ground truth). This hyperparameter might be related to the firing rate of the indicators used in the training dataset. We observe that a window of (11, 5) suits best for the spikefinder challenge dataset. There was a little improvement for all of the evaluation measures upon providing a smoothed Gaussian as the target. The difference in performance was 0.1% for the correlation measure in the spikefinder test set and around 12% for the training set. Even with a non-smoothed (“actual”) ground truth, the system’s overall performance is better than the baseline methods (Figs 2 and 3 (***left***)).

In order to further validate the effect of windowing, we performed 10-fold cross-validation of the spikefinder dataset and reported the performance in Table 6. Five training sets and five test sets were used for the leave-one-dataset-out cross-validation. The Gaussian window-based smoothing of the discrete spike target helped improve the correlation and rank measures by 5% and 7.5%.

**Table 6:** Results on 10-fold cross-validation with and without Gaussian windowing for the training target. GT: Ground Truth, AVG: Average and STD: Standard Deviation.

Measure	GT AVG ± STD	Gaussian AVG ± STD	*δ* AVG(%)
Correlation	0.781 ± 0.059	0.831 ± 0.055	5.0
Rank	0.647 ± 0.061	0.722 ± 0.064	7.5
AUC	0.879 ± 0.019	0.876 ± 0.018	-0.3

#### 2.4.2 Architecture

**Fig 4:**
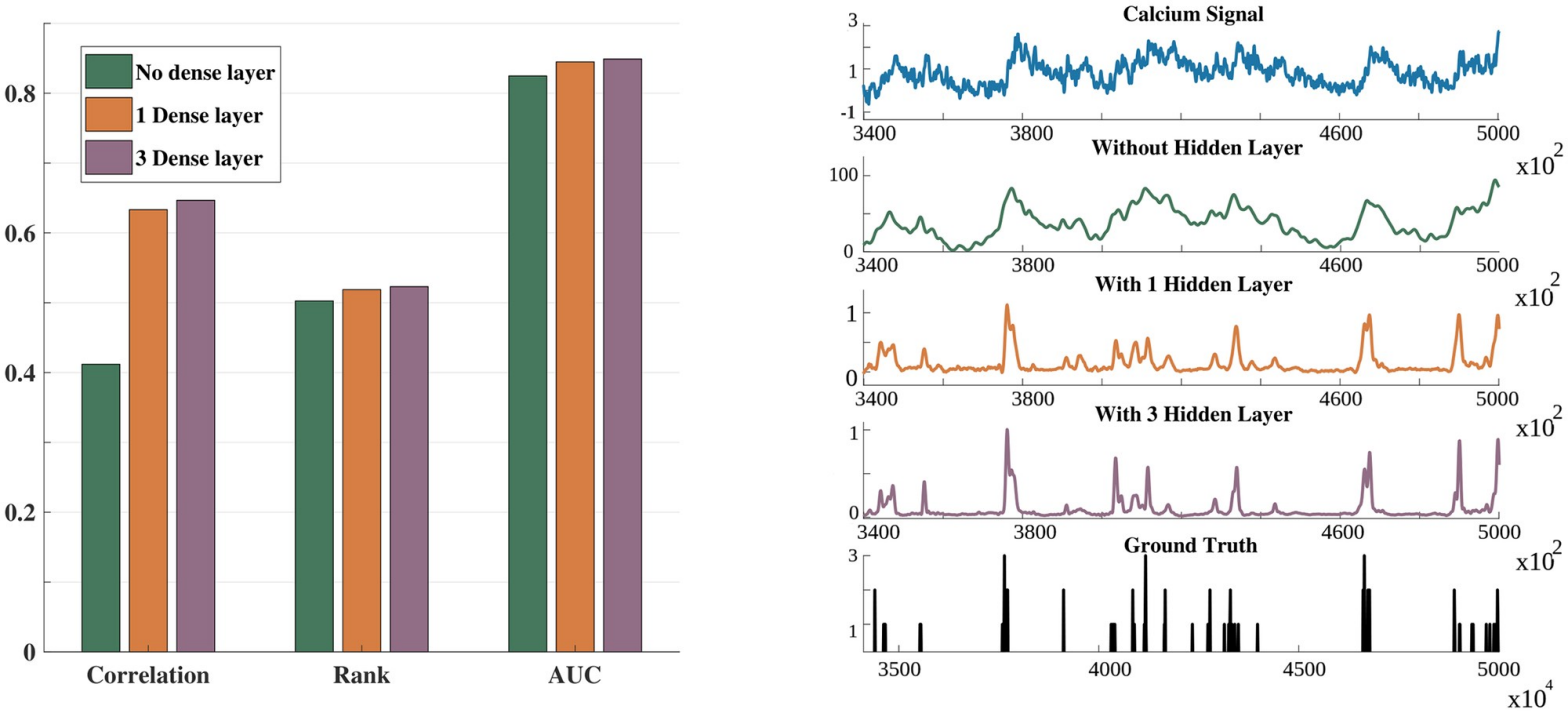
Evaluation measures with changes in number of hidden layers. (***left***) Bar diagram depicting the difference in correlation, rank and AUC when no hidden layer, 1 hidden layer and 3 hidden layers are used in the S2S network, respectively. (***right***) Illustrative example showing the improved spike estimates when 3 hidden layers are used compared to one hidden layer.

The number of hidden layers in the network architecture (explained in the Section 2.4.2) was chosen based on cross-validation. We examined the role of the hidden layers by varying the number of hidden layers from three (used in the experiments reported in the Results part) to zero. The target for training was obtained by convolving the Gaussian window (11, 5) with the discrete spike signal. Fig 4 presents the results in terms of the three evaluation measures. It can be observed that, even without a hidden layer, the proposed S2S method yielded a system that was competitive to state-of-the-art systems. The performance improved with the addition of hidden layers. We found that the performance was saturated beyond three hidden layers. As illustrated in the subplot with an example of the input signal, S2S with the hidden layer(s) improves the denoising and spike-resolving capabilities compared to the S2S without a hidden layer.

#### 2.4.3 Generalization ability

**Fig 5:**
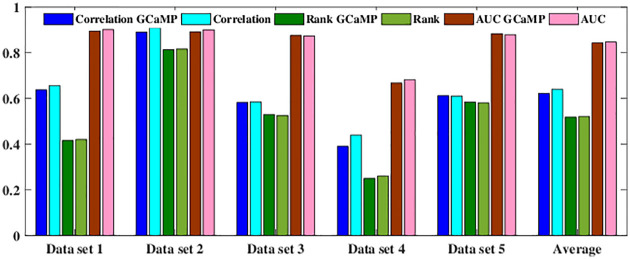
Dataset-wise performance showing the generalization ability of the three hidden layer S2S network. “GCaMP” indicates that the training was done only with GCaMP indicator dataset. Datasets 1, 2 and 4 are based on OGB indicator.

The spikefinder challenge consisted of data obtained with two different indicators, namely, GCaMP indicator and OGB indicator. In the challenge protocol, both the training and test conditions contained signals from these indicators. We examined the proposed approach’s generalization ability across different indicators by training the S2S only on the GCaMP indicator data in the training set and testing on the test set of spikefinder contest containing data from both indicators. More specifically, the *invariance* of the spikefinder test set performance to a change in the training set was considered. We trained S2S with one hidden layer and three hidden layers. The training was carried out with targets obtained by convolving the ground truth discrete spike signals with Gaussian window (11, 5). Table 7 presents the results for both one and three hidden layer S2S. It was observed that the S2S trained only with the GCaMP indicator yielded competitive performance with that trained using all the training data. Furthermore, it was observed in Fig 5 that the evaluation measures on OGB datasets only gracefully degraded for the GCaMP-only trained model in comparison with the combined-model for *all* the datasets.

**Table 7:** Generalization across indicators. Experimental results with various number of hidden layers.

Configuration	Training Indicator(s)	Correlation	Rank	AUC
1 hidden layer	GCaMP	0.608	0.510	0.832
1 hidden layer	GCaMP + OGB	0.639	0.519	0.845
3 hidden layers	GCaMP	0.622	0.518	0.843
3 hidden layers	GCaMP +OGB	0.640	0.521	0.847

We extended the experiments further by considering only one indicator for the training and the other for testing. For training a GCaMP-only model, we used two training datasets in the spikefinder challenge and the five optional datasets provided for training. For training the OGB-only model, the datasets from both train and test splits of spikefinder challenge were taken. This resulted in six OGB datasets for training and two GCaMP datasets for testing. The results of the experiments are given in Table 8.

**Table 8:** Results on generalization experiments. **Experiments 2b and 3b represents generalization from GCaMP to OGB and vice-versa**. For every experiments, the number of datasets used for training and testing are given inside the brackets.

Sl No.	Experiment	Correlation	Rank	AUC
1a	Train full (10) & Test full (5)	0.640	0.522	0.848
1b	Train on GCaMP (7) & Test full (5)	0.635	0.516	0.838
2a	Train full (10) & Test on OGB (3)	0.672	0.498	0.827
2b	Train on GCaMP (7) & Test on OGB (3)	0.656	0.488	0.811
3a	Train full (10) & Test on GCaMP (2)	0.594	0.558	0.881
3b	Train on OGB (6) & Test on GCaMP (2)	0.465	0.493	0.803

The results corresponding to rows 1a and 1b in Table 8 considered the performance invariance of S2S on the spikefinder challenge test set with a GCaMP-only based training. It was observed that there had a drop of 0.5% in the primary evaluation measure. Rows 2a and 2b correspond to the differences in OGB performance when only the GCaMP is used for the training. The reduction in performance was 1.6%, 1% and, 1.6%, respectively, for correlation, rank, and AUC. A somewhat different observation was made for generalization performance for OGB indicators (Rows 3a and 3b in Table 8). The absolute difference in correlation, rank, and AUC were 13%, 6%, and 8%, respectively.

From the results, it was observed that S2S is generalizable from GCaMP to OGB. However, similar conclusion can not be drawn in the case OGB to GCaMP. The primary reason is the absence of large amount of data for OGB.

#### 2.4.4 Layer-wise output

**Fig 6:**
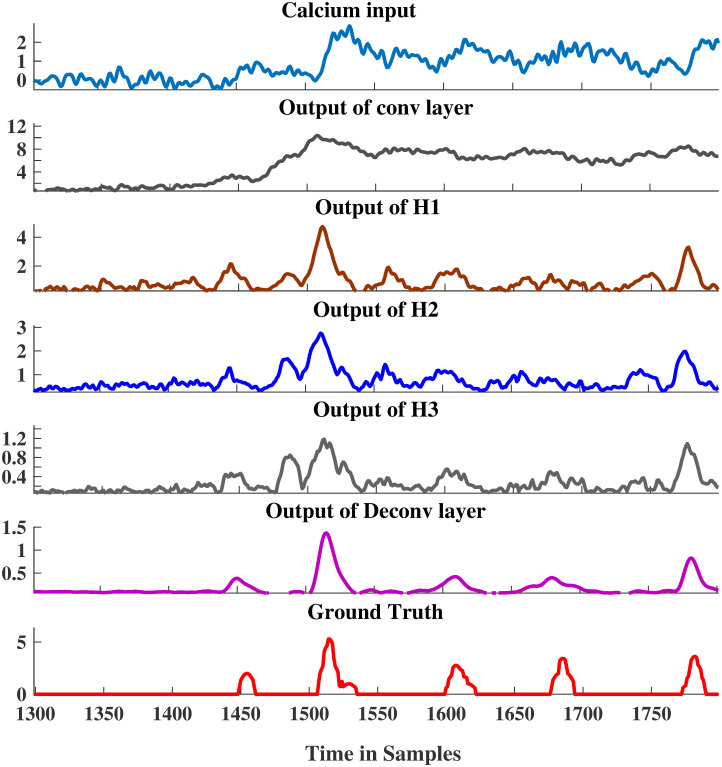
Layer-wise output of a 3-dense layer S2S. Note that the calcium changes which are not clearly distinguishable in the original fluorescence signal are amplified in the output of the deconvolution layer.

The S2S method is interpretable, in the sense that the processing carried out by each layer in the network can be visualized to gain insights. Fig 6 shows the layer-wise outputs of the three hidden layer architecture. These outputs were generated by feeding the calcium input signal and calculating the total response per layer with each layer’s trained weights. For more details regarding the estimation of layer-wise output, the reader is advised to refer to the Methods section (Section 4). For the given calcium fluorescence input, the analysis convolution layer seemed to make it less-noisy (or smooth out slight variation in the signal), while preserving possibly the information “relevant” for spike estimation. Observe that the shape of the input signal is preserved. The first hidden layer output after ReLU non-linearity seemed to resolve potential spike positions. This output was then refined or filtered by the subsequent hidden layers and the output synthesis layer by enhancing the signal at the spike locations and suppressing the spurious spike locations. This yielded an output spike signal estimate that correlates well with the ground truth. Each layer contributed towards maximizing the similarity or correlation between the spike signal estimate and the ground truth. It was worth noting that the output of hidden layers H2 and H3 did not differ too much when compared to H1. The output synthesis convolution layer carried out the significant refinements. This observation was in line with the earlier observation that one hidden layer S2S yields performance comparable to the three hidden layer S2S.

#### 2.4.5 Filter responses

**Fig 7:**
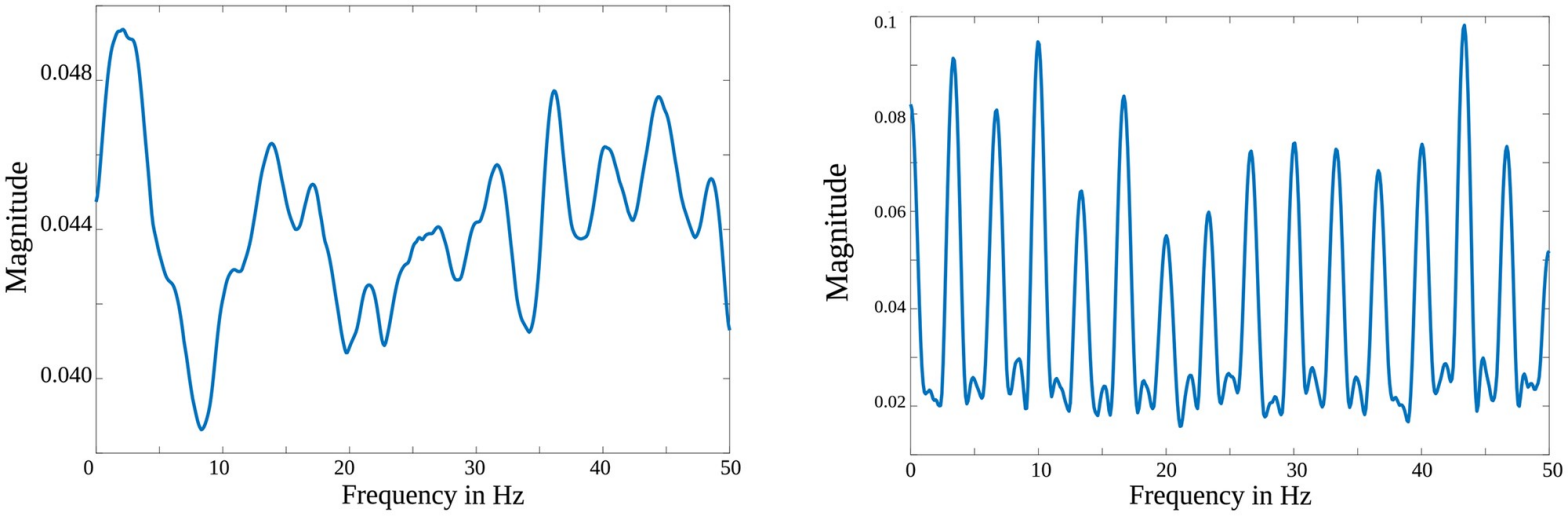
Cumulative frequency responses of filters. (***left***) Shows the frequency response of the analysis filters. (***right***) Shows the response of synthesis filters having larger amplitudes at equal frequency intervals. The energy at the output of S2S is purely concentrated on spikes both at the temporal and the spectral domain.

The S2S method learned to synthesize the spike information from the calcium fluorescence signals due to the filters in the analysis and synthesis convolutional layers. Hence, we analyzed the frequency response of the filters. Fig 7 shows the cumulative frequency response of the filters in the analysis convolutional layer and the synthesis convolutional layer. It was observed that the analysis layer gives emphasis to low-frequency information (between 0-5 Hz). In the case of the synthesis layer, we observed a harmonic structure, which could be attributed to the fact that the layer predicts the spike signal. The harmonic structure was present in the time-domain analysis as well. This is probably because the network was learned to predict spike trains with different time-scales and frequency components. The de-convolution layer that synthesized the signal at the output makes our approach distinct. It was more effective than the neural network-based methods that estimated spikes in a sample-by-sample manner (refer to Section 2.3).

## 3 Discussion

We showed that the proposed S2S method could yield state-of-the-art results in the spike estimation task. Our method resulted in significant improvement in both primary and secondary evaluation measures. This architecture seems to be appropriate for the spike estimation problem. The synthesis layer (which reconstructs the spike signal) and the cost function based on the correlation coefficient resulted in faster convergence and better performance.

### 3.1 Nature of improvement

**Fig 8:**
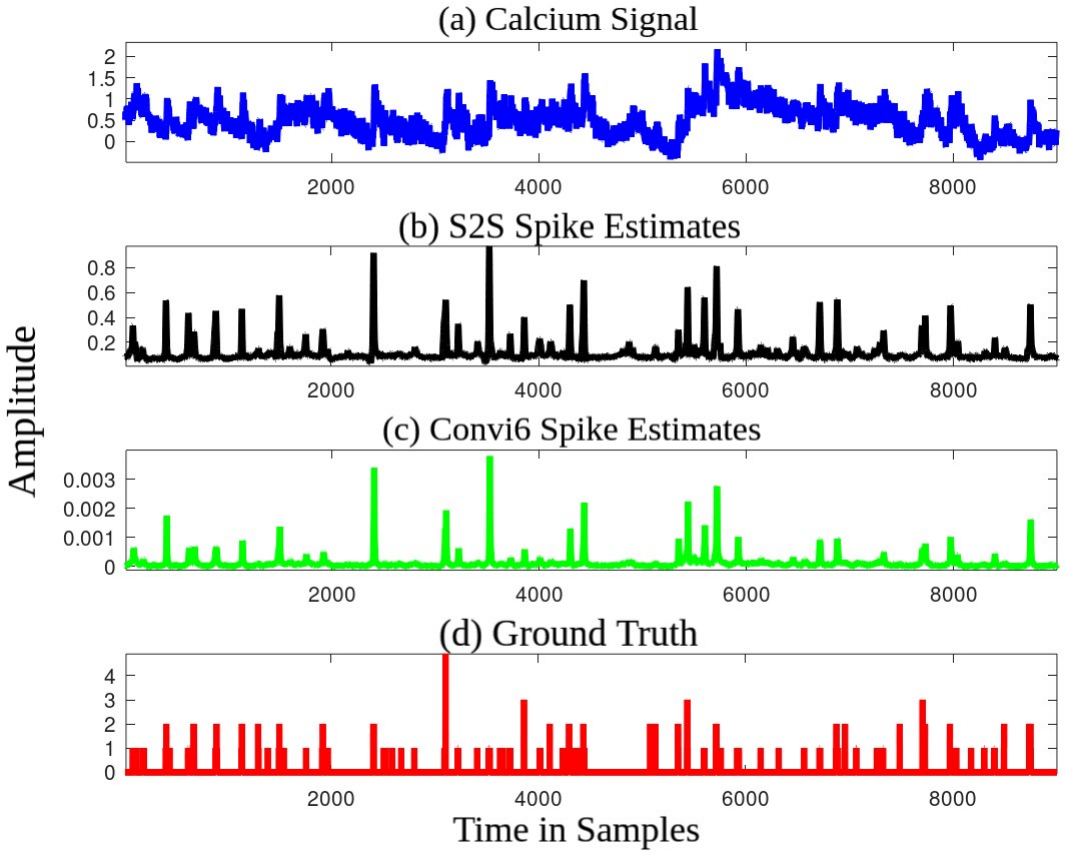
Comparison of spike estimates. (a) An example calcium input signal, spike estimates of (b) S2S and (c) convi6 methods, and (d) discrete ground truth. Observe the similarity in shape between the S2S and the ground truth, compared to the convi6 method.

S2S has significantly better linear and non-linear correlation measures and a comparable AUC value to that of the baseline algorithms. We further observe that these differences are preserved even at a higher resolution (10 ms bin width) as well (refer to the scalability experiments in Section 2.3). Hence, S2S is able to infer spike trains (100 Hz) as well as the firing rates (25 Hz). Fig 8 compares the spike estimates obtained from the S2S and the baseline convi6 method to the discrete ground truth at 40 ms bin width.

It can be observed that the peak locations are better resolved in S2S than the convi6. The dynamic range of S2S is much larger than the baseline, and this is reflected in the correlation measure. For every action potential, the convi6 has a very small increase in the amplitude of the estimated signal, typically around 0.001. The corresponding increase in the amplitude of the S2S spike estimate is 0.2-0.3. The amplitude of the individual S2S spike estimates are especially high when the actual spike count is greater than one or when a burst of spike occurs, similar to the ground truth.

The convi6 estimates have a shorter dynamic range than that of the S2S estimates. To confirm that, we compute the average deviation of the amplitude between the spike estimate and the ground truth. The average deviation of the S2S spike estimate with respect to the ground truth is much smaller than that of the convi6 method. The average deviation varies from 0.02 to 0.45 per test file for the S2S spike estimate, whereas it varies from 0.55 to 1.48 per test file for the convi6 spike estimate. To further validate the statistical significance of the deviation per test file, we conduct a pair-wise t-test considering all the 32 test files. The null hypothesis (H) is that there is no statistical significance in the deviation per test file. The null hypothesis is rejected with a very high confidence(*α* = 3.1E-30). We also observe that the improvement in performance of S2S method is independent of Gaussian windowing that is applied optionally to the discrete spike estimates during the training. The S2S output amplitude is proportional to that of the ground truth across datasets, as evident in the correlation measures. The threshold for getting the spike train can be different depending on the dataset. The dynamic range is wide enough to put multiple thresholds to differentiate the occurrences of one or more spikes at a time. This is an important advancement over the previous spike estimation methods.

In Fig 8, it can be observed that the relative spike positions at the output of S2S are similar to that of convi6, as also pointed out by the AUC measure. Hence, the improvement in linear and non-linear correlation measures can be mainly attributed to the amplitude of the spike estimates, which are more accurate and quite useful for further thresholding.

### 3.2 Design aspects of the S2S method

We examined various design aspects of the S2S method. Our studies revealed that: (a) making the targets less sparse by convolving the ground truth discrete spike signal with Gaussian window helps, (b) at least one hidden layer is needed to resolve the spike locations, (c) the method is not sensitive to initialization; converges within 50 epochs, and yields similar performances when retrained (the results are reproducible), and (d) the method is capable of generalizing across unseen indicators.

One of the major concerns in using a supervised approach for spike inference is its computational complexity. Generative approaches, on the other hand, require mainly novel parametric settings for new datasets. S2S method, although being a supervised method, is computationally efficient. All the top-performing supervised algorithms in the spikefinder contest had recurrent units in the architecture, which resulted in increased training time. The proposed S2S system is 50 times faster than the best performing supervised model when trained with GPU using Sun Grid Engine, potentially because the network has very few trainable parameters and the use of time-distributed hidden layers. As presented in the Methods section, the three hidden layer S2S has only 8790 trainable parameters.

### 3.3 Multiple evaluation measures

Performance evaluation of a spike estimation algorithm should consider the spike estimate’s overall shape, monotonic relationship with the ground truth, and the estimates’ accuracy with multiple thresholds. There is no unique measure that encompasses all this information [35]. For instance, the spike train is considered as a density function, and its shape is evaluated with the Pearson’s correlation coefficient. However, this measure is invariant under affine transformations. Thus, it is not easy to interpret the outputs as spike counts or rates. The Spearman’s rank-order correlation coefficient evaluates both the strength and the direction of the association between the two ranked variables. The dynamic ranges of the spike information and discrete spike train are employed for ranking. Unlike the Pearson’s correlation coefficient, this measure considers the non-linear relationship between the variables. Finally, the area under the receiver operating characteristics (AUC) measures how well the spikes have been detected. AUC is not sensitive to changes in the relative height of different parts of the spike information. Thus, this measure alone is not adequate. The proposed S2S method outperforms all the other systems in terms of Pearson’s correlation coefficient and Spearman’s rank correlation coefficient and achieves similar performance in AUC (only the DeepSpike method has marginally high AUC). This indicates that the S2S is yielding a better estimate of spike signal than existing generative and supervised methods. Specifically, the S2S method resulted in an improvement of 46% in primary evaluation measure compared to the STM baseline, which is two times that achieved by the Spikefinder contest (23%). This performance gain could help in further directions in spike estimation based on signal reconstruction strategies.

## 4 Methods

### 4.1 Signal-to-signal neural network for spike signal estimation

As discussed earlier in the Introduction section (Section 1), approaches are emerging to convert one sequence into another sequence in an end-to-end manner in various fields related to sequence processing such as speech processing and natural language processing. For instance, converting a sequence of acoustic features to a sequence of letters or words and translating the sequence of words from one language to another language. We formulated the spike estimation problem from the calcium fluorescence signal as a sequence-to-sequence conversion problem, as a signal is a time-ordered measurement. Towards that, we took inspiration from recent work on end-to-end speech source separation [27] to develop a signal-to-signal network which was then learned to predict/estimate spike signal given the calcium fluorescence signal as input (as illustrated in Fig 1). Intuitively, the S2S method can be seen as a single-channel signal enhancement system where the desired spike signal present in the calcium fluorescence signal was enhanced, while the undesirable signals or variabilities were suppressed.

#### 4.1.1 S2S Architecture

The S2S method consisted of a convolution layer followed by a fully connected dense layer(s) and an output convolution layer. The hidden layers were implemented in a time-distributed manner, i.e., the output of the first convolution layer for every segment of the calcium fluorescence signal was independently processed by the hidden layer(s) and then synthesized by the output convolution layer. In the network, the output of each hidden node or filter was fed to a rectified linear unit (ReLU) non-linearity, except for the output convolution layer. ReLU units in the neural network did not saturate unlike *sigmoid* and *hyperbolic tangent* activations. They also helped in learning a non-negative representation in the successive layers.

The hyper-parameters of the S2S system were: the length of signal input *w*_*seq*_, kernel width *kW*_*in*_ and kernel shift *dW*_*in*_ of the input convolution layer, number of filters in the input convolution layer *nFilt*_*in*_, number of hidden layers *I* and the number of nodes *nhu*_*i*_ in each hidden layer *i* ∈ {1, …*I*}, kernel width *kW*_*out*_ and kernel shift *dW*_*out*_ of the output convolution layer and the number of filters in the output convolution layer *nFilt*_*out*_. Based on the systems reported in the spikefinder challenge, we set *w*_*seq*_ = 100 samples (i.e., 1 sec). We set *kW*_*in*_ = *w*_*seq*_ and *dW*_*in*_ = 1 sample. In the output convolution layer, *kW*_*out*_ = *nhu*_*I*_ (i.e. number of nodes in the last hidden layer), *dW*_*out*_ = 1 sample and *nFilt*_*out*_ = *w*_*seq*_. In other words, for every frame of 100 sample input, the output convolution layer synthesized 100 samples of spike signal, which were then overlapped and added to get a spike signal of the same length as that of the input calcium fluorescence recording. The number of frames was determined by *dW*_*in*_, which was one sample. The remainder of the hyper-parameters *nFilt*_*in*_ = 30, *I* = 3 and *nhu*_1_ = *nhu*_2_ = *nhu*_3_ = 30 were determined by cross-validation on the training set through a coarse grid search. Thus, the three hidden layer S2S compared against the state-of-the-art methods in the Results section had (100 × 30 + 30)+ (30 × 30 + 30)+ (30 × 30 + 30) + (30 × 30 + 30) + (30 × 100) = 8790 parameters. In the case of the one hidden layer study presented in the Analysis section, the network had (100 × 30 + 30) + (30 × 30 + 30) + (30 × 100) = 6960 parameters, and in the case of no hidden layer (100 × 30 + 30)+ (30 × 30) = 6030 parameters.

#### 4.1.2 Training the S2S system

We trained the S2S system by maximizing the Pearson’s correlation coefficient between the spike signal estimated by the network and the ground truth spike signal. We also investigated other cost functions such as mean square error and cross-correlation with sigmoid non-linearity. Pearson’s correlation coefficient yielded the best system on both the cross-validation experiments conducted on the training set and the test set. For training the S2S, we split the training data in the spikefinder challenge into two parts: a training set (80%) and a validation set (20%). At each training epoch, the training set was used for training the network parameters, and the validation set was used for cross-validating the network. We used Adam optimizer with a starting learning rate of 0.001 and a batch size of 20. Early stopping with a patience factor of 6 was used to stop the training whenever the validation loss increases compared to the previous epoch. As the ground truth was a sparse discrete signal, the target spike signal was optionally convolved with a Gaussian window to make the targets less sparse for the network’s efficient training. The Gaussian window (11, 5) was obtained through cross-validation, i.e., the window that yielded the best validation cost. The implementation was carried out on Keras [36] with TensorFlow [37] backend. The software based on s2s is available at https://github.com/Jiltseb/S2S_for_spike_inference.

### 4.2 Data

We used the spikefinder challenge dataset to validate the proposed S2S method. We followed spikefinder evaluation [19] owing to the following reasons: First, it is one of the largest publicly available dataset containing different scanning rates and methods and calcium indicators. Second, the spikefinder challenge provided a benchmarking framework to compare different spike estimation methods using different evaluation measures. Finally, the challenge provided state-of-the-art baseline systems to which the proposed method can be compared to. As mentioned earlier, the spikefinder dataset had five benchmarking datasets consisting of 92 recordings from 73 neurons. Five additional datasets from GENIE project [32] have been provided to ensure that the supervised models do not over-fit to the training data and to test the generalization ability. The zoom factor of all the recordings was at use-case resolutions for the calcium imaging experiments. For further information, the reader is referred to [19]. The dataset is available at https://github.com/codeneuro/spikefinder.

### 4.3 Comparison framework

The proposed method’s performance was compared to two sets of baselines: The results were first compared to the top-five algorithms in the spike finder contest. They are either generative [12, 14] or supervised [17, 19, 33, 34] approaches. The generative approach, which was initially published in [12] use a biophysical model and estimates the maximum probable spike information from the fluorescence signals. Efficient Bayesian inference was performed on this MLSpike model, including a drifting baseline and non-linear modeling of calcium to fluorescence conversion. MLspike was the winner of the spikefinder contest. This algorithm’s performance was boosted (with respect to the original work) owing to the parameter tuning with respect to the challenge dataset. The second best generative approach [14] was based on an auto-regressive approximation to the calcium fluorescence signal. Spike information was then estimated by solving a non-negative sparse optimization problem. Based on the advances in neural network-based models for various applications, it was not surprising that 7 out of top-10 algorithms are deep learning-based supervised algorithms (refer to Table 1). All the top-performing algorithms used recurrent layer in the network. For the best-performing algorithms, we have taken the results from the spikefinder challenge [19], and by running the evaluation scripts (https://github.com/berenslab/spikefinder_analysis).

We compared the proposed network’s performance and efficiency with the best-performing supervised baseline that is available as an open-source Python software (https://github.com/kleskjr/spikefinder-solution). We trained the network using open-source software. However, the model weights provided by the “convi6” authors yielded better linear correlation values than those obtained by training the neural network. Convolutional layers take a broad temporal context to predict a single output corresponding to the spike information. The dataset index that was provided as an auxiliary input to the network observed to improve the system’s performance. Convolutional layers are followed by an intermediate LSTM-recurrent layer and further by convolutional layers of smaller width. The learned filters collectively capture the spike-related information.

### 4.4 Evaluation

All the evaluations were done at 25 Hz (40 ms bin width), following the protocol used in spikefinder evaluations (https://github.com/berenslab/spikefinder_analysis). As per the protocol, we used Pearson’s correlation coefficient as the primary evaluation measure. Δ correlation is computed as the average difference in correlation coefficient compared to the STM algorithm (refer to Table 2). The secondary evaluation measure was Spearman’s rank correlation coefficient, which considers the direction and non-linear relationship between the estimates and actual spikes. The area under the ROC curve (AUC) curve was the final measure, which evaluates detection of spikes in a 40 ms bin. We used the function *roc_curve* from the *scikit-learn* [38] Python package for computing the AUC and *spearmanr* function from the *scipy* [39] Python package for computing the rank. These evaluation measures collectively denote the similarity with the ground truth. We considered them in the order of their preference as in the spikefinder challenge. The test set’s rank measure was not included in the challenge paper, although it was the secondary measure on the training sets.

## 5 Conclusion

This paper presented a neural network-based signal-to-signal conversion approach (S2S) for spike estimation from imaging data. The neural network analyses and filters the raw calcium imaging data at the input and synthesizes or estimates the spike signal at the output, in an end-to-end manner. During training, the parameters of the neural network are estimated by a cost function based on Pearson’s correlation coefficient between the estimated spike signal and the ground truth. Evaluations on the Spikefinder benchmarking data set showed that the proposed approach achieves state-of-the-art performance in the spike estimation task. An exhaustive set of experiments were performed to evaluate the statistical significance, scalability and generalizability of the results and learning capacity of hidden layers.
